# Fucosidosis in Tunisian patients: mutational analysis and homology-based modeling of FUCA1 enzyme

**DOI:** 10.1186/s12920-021-01061-3

**Published:** 2021-08-23

**Authors:** Latifa Chkioua, Yessine Amri, Chayma Saheli, Ferdawes Fenni, Hela Boudabous, Hadhami Ben Turkia, Taieb Messaoud, Neji Tebib, Sandrine Laradi

**Affiliations:** 1 Research Laboratory of Human Genome and Multifactorial Diseases, Faculty of Pharmacy, University of Monastir, Street Avicenne, 5000 Monastir, Tunisia; 2 Biochemistry Laboratory (LR 00SP03), Bechir Hamza Children’s Hospital, Tunis, Tunisia; 3 Pediatrics Department, La Rabta Hospital, Tunis, Tunisia; 4 The Auvergne-Rhône-Alpes Regional Branch of the French National Blood System EFS/GIMAP-EA 3064, 42100 Saint Etienne, France

**Keywords:** Fucosidosis, Alpha-l-fucosidase (FUCA1), Angiokeratoma, Bioinformatics tool, Mutations

## Abstract

**Background:**

Fucosidosis is an autosomal recessive lysosomal storage disease caused by defective alpha-l-fucosidase (FUCA1) activity, leading to the accumulation of fucose-containing glycolipids and glycoproteins in various tissues. Clinical features include angiokeratoma, progressive psychomotor retardation, neurologic signs, coarse facial features, and dysostosis multiplex.

**Methods:**

All exons and flanking intron regions of FUCA1 were screened by direct sequencing to identify mutations and polymorphisms in three unrelated families with fucosidosis. Bioinformatics tools were then used to predict the impacts of novel alterations on the structure and function of proteins. Furthermore, the identified mutations were localized onto a 3D structure model using the DeepView Swiss-PdbViewer 4.1 software, which established a function-structure relationship of the FUCA1 proteins.

**Results:**

Four novel mutations were identified in this study. Two patients (P1 and P2) in Families 1 and 2 who had the severe phenotype were homoallelic for the two identified frameshift mutations p.K57Sfs*75 and p.F77Sfs*55, respectively. The affected patient (P3) from Family 3, who had the milder phenotype, was heterozygous for the novel missense mutation p.G332E and the novel splice site mutation c.662+5g>c. We verified that this sequence variation did not correspond to a polymorphism by testing 50 unrelated individuals. Additionally, 16 FUCA1 polymorphisms were identified. The structure prediction analysis showed that the missense mutation p.G332E would probably lead to a significant conformational change, thereby preventing the expression of the FUCA1 protein indeed; the 3D structural model of the FUCA1 protein reveals that the glycine at position 332 is located near a catalytic nucleophilic residue. This makes it likely that the enzymatic function of the protein with p.G332E is severely impaired.

**Conclusion:**

These are the first FUCA1 mutations identified in Tunisia that cause the fucosidosis disease. Bioinformatics analysis allowed us to establish an approximate structure–function relationship for the FUCA1 protein, thereby providing better genotype/phenotype correlation knowledge.

**Supplementary Information:**

The online version contains supplementary material available at 10.1186/s12920-021-01061-3.

## Background

Fucosidosis (OMIM ≠ 230000) is a rare autosomal recessive lysosomal storage disease caused by a deficiency of the alpha-l-fucosidase enzyme (FUCA1, EC 3.2.1.51; 612280). This enzyme hydrolyzes fucose at the non-reducing end of glycolipids and glycoproteins in various tissues, including the liver, spleen, and heart [1].

The human alpha-l-fucosidase gene (612280) spans 23 kb in length, contains eight exons and seven introns, and is mapped to the region 1p34.1–1p36.1 [2]. The 2053-bp full-length cDNA encodes a signal peptide of 22 amino acids and a mature protein of 439 amino acids. The compiled cDNA sequence of FUCA1 consists of 2053 bp. It comprises a 5′untranslated sequence, a 1383-bp open reading frame, and a polyadenylation signal AATAAA [2, 3].

Patients with fucosidosis were classified as having severe and attenuated forms of the disease, depending on the degree of the observed psychomotor regression. The severe form, appearing between 6 months and 1 year of age, is characterized by an early onset of psychomotor retardation, severe motor degeneration, severe neurologic deterioration, and death within the first decade of life. The attenuated form is characterized by milder psychomotor retardation and neurologic signs, the development of angiokeratoma corporis diffusum, and longer survival [4, 5].

To date, 30 mutations in the *FUCA1* gene associated with various clinical phenotypes of fucosidosis type I have been described (Human Gene Mutation Database, http://www.hgmd.cf.ac.uk, accessed in June 2019) [6].The spectrum of FUCA1 mutations includes seventeen missense/nonsense mutations, six small deletions, three large deletions, two splice-site mutations, one small insertion, and one duplication. These mutations are mostly located in the glycoside hydrolase domain (catalytic domain, amino acid residues 35–370) and the C-terminal domain (amino acid residues 372–463) of alpha-l-fucosidase, resulting in nearly absent enzymatic activity [7].

This study is the first attempt to provide an accurate template of the FUCA1 3D structure in order to predict the effect of the newly identified Tunisian mutations on the FUCA1 structure and function. The previously reported mutations were then analyzed and localized onto the generated structure to determine the crucial residues or domains for normal protein function and secretion.

## Patients and methods

### Ethics statement

**Fig. 1:**
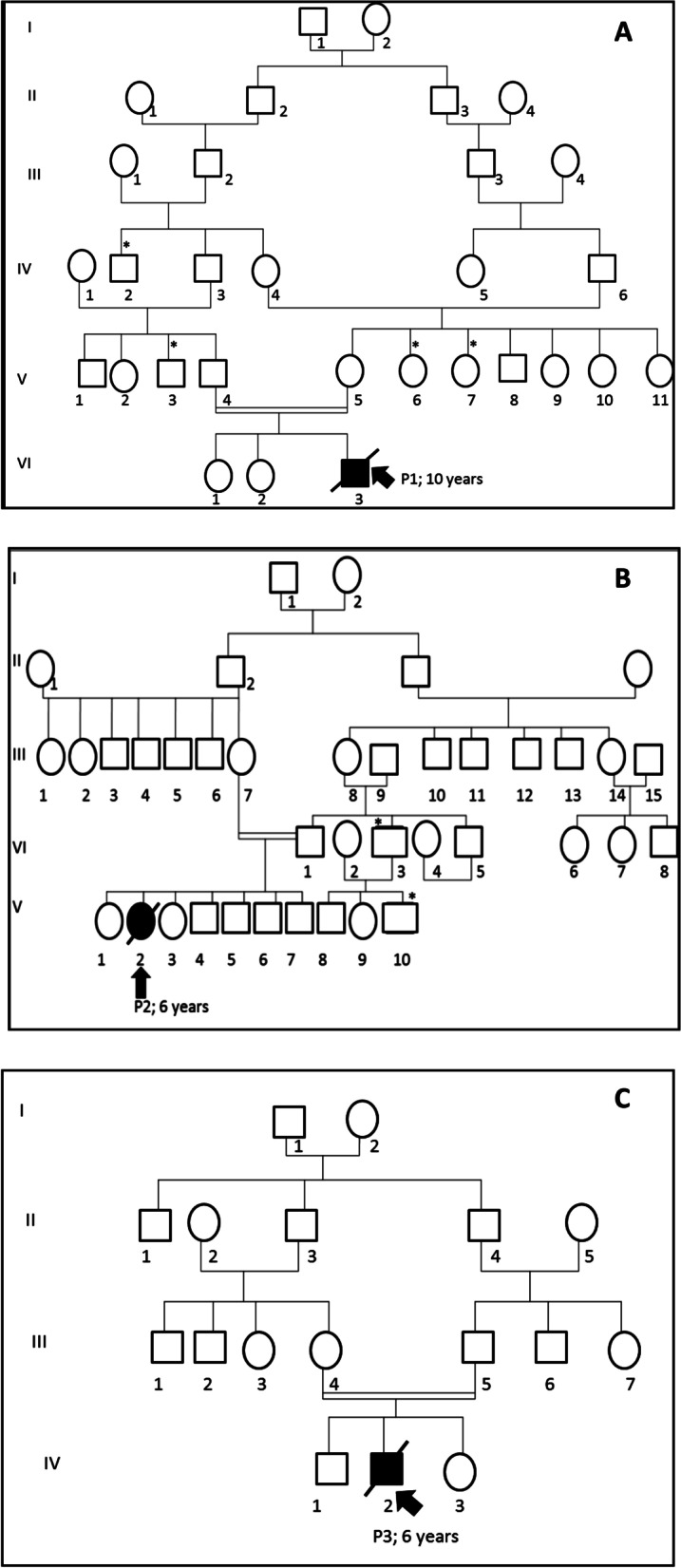
Pedigrees of the three investigated fucosidosis Tunisian families (**A**–**C**). Squares and circles indicate male and female members, respectively. The arrow denotes affected individuals. Double lines indicate consanguineous matings. The asterisk indicates family members with clinical history fucosidosis

This study was carried out on three patients (P1, P2, and P3) with fucosidosis, diagnosed between 2007 and 2010 in the pediatric department of La Rabta Hospital, Tunisia. Each case was classified as type I or II. All investigated patients were offspring of consanguineous marriages between first and second cousins from different areas of Tunisia (Fig. 1).

Family histories and main clinical data are reported in Table 1. All the affected children had healthy siblings.

**Table 1:** Family history and description of the three fucosidosis patients

Features	Patient P1	Patient P2	Patient P3
Consanguinity of parents/degree	First degree	Second degree	Second degree
Age of diagnosis (Yr/Mo)	18 Mos	12 Mo	24 Mo
Age of onset (Yr/Mo)	9 Mo	8 Mo	18 Mo
Age of death	10 Yr	6 Yr	6 Yr
Sex	Male	Female	Male
FUCA1 assay (nmol/mg prot)	0.00	0.00	2.00
	18.4 (2.3–41.9) in control subject		
Elements excreted in the urine	Fuc(α1-6)GlcNAcβ4-Asn++++; Fucosylated oligosaccharides++++		
Neurological deterioration	++++	++	+
Mental retardation	+++	+++	+++
Growth retardation	− 6SD	− 4SD	− 4SD
Macroglossia	++	++	+
Spasticity	+++	+++	++
Axial hypotonia	+++	+++	++
Visceromegaly	Hepatomegaly	–	–
Recurrent respiratory infections	+++	+++	++
Skin abnormalities	–	Angiokeratoma	Angiokeratoma
Type of fucosidosis	Type I	Type I	Type II
FUCA1 mutations identified	p.K57Sfs*75/p.K57Sfs*75^a^	p.F77Sfs*55/p.F77Sfs*55^a^	p.Gly332Glu /C.662+5g>c^a^
Polymorphisms/sequence variants of the FUCA1 gene	p.P213P;(c.639A>T)^a^+/+	p.Y216F;(c.647A>T)+/+	rs1215568236+/−, p.P10R+/−, rs370615681+/+, rs1329117558+/−, rs965877153T+/, rs1344267327+/+
	IVS3-25T>A; (c.647A>T)^a^+/+	p.L194N^a^+/+	
	IVS2-110inst;(c.524 -110inst)^a^+/+	p.P10R +/−	
	IVS2+38c>g; (c.509+38 c>g)^a^+/+	IVS 3–29 a>c;(c.647–29 a>c)^a^+/+	
	rs180788085 +/−, rs907245739+/+, rs1329117558+/+	IVS2-78 del t;(c.647-78delt)^a^+/+	
		p.L172L+/+	
		rs1329117558 +/−, rs370615681 +/−	

Yr, Year; Mo, Month; +/+, homozygous state; +/−, heterozygous state

^a^Novel sequence variants

This study was approved by the Ethics Committee of the La Rabta Hospital in Tunisia since 2010, and the families provided informed consent prior to collecting blood samples. All procedures were in accordance with the ethical standards of the responsible committee on human experimentation (institutional and national) and with the Helsinki Declaration of 1975, as revised in 2000 and approved by the Ethics Committees of the respective Tunisian hospitals.

### Family 1/patient 1 (P1)

He was the third child of healthy second-degree consanguineous Tunisian parents (Fig. 1A), originating from the North of Tunisia (Testour). He was eutrophic at birth, and during the first year of life, his psychomotor and mental characteristics were perfectly normal. The diagnosis was performed at the age of 18 months when he had progressive walking difficulty with a tendency to fall. At the age of nine months, the first symptoms of the disease appeared with episodes of recurrent broncho-pneumopathies. Clinical examination detected hepatomegaly, macroglossia, and slight facial dysmorphism with severe growth retardation. At the age of one year, the child developed a psychomotor regression associated with stunting. Neurological involvement worsened to an array of spastic quadriplegia, loss of communication, blindness, and epilepsy.

The patient’s phenotype was classified as fucosidosis type I because of the rapid progression of neurological deterioration, the absence of angiokeratoma, and survival that did not exceed the first decade of life.

### Family 2/patient 2 (P2)

She was the third child born in a consanguineous family, and she had six brothers unaffected by fucosidosis (Fig. 1B). She originated from the central West of Tunisia (Sbeïtla). Family history was positive for an uncle with mental retardation and a younger cousin with the same symptoms.

At eight months of age, the girl was referred to the pediatric department of La Rabta Hospital of Tunis (North of Tunisia) with psychomotor retardation, loss of smile response, and sitting station. Ten months later**,** she was evaluated for facial dysmorphism, convergent strabismus, gingival hypertrophy of angiokeratomas, and angiokeratomas under the nails. She died due to cardiorespiratory complications when she was six years old.

### Family 3/patient 3 (P3)

He was the second child born to consanguineous parents; he had three healthy brothers and a negative familial history of fucosidosis (Fig. 1C). He had an early onset of psychomotor retardation within the first year of life. He had developed severe spastic quadriplegia at 18 months of age. Mild coarse facies were noted at the age of two years, and severe growth retardation and angiokeratoma were noted at the age of three.

### Biochemical assay

The leukocyte FUCA1 activity was measured at the Biochemistry Laboratory of Cochin Hospital, Paris, France using the synthetic fluorigenic substrate, 4-methylumbelliferyl α-l-fucoside [8].

### Mutation screening

The leukocytes of patients were stored at − 20 °C since 2010, then the genomic DNA was isolated from leukocytes of patients with type I fucosidosis according to the standard salting out procedure [9]. The DNA was used as a template for PCR amplification of the *FUCA1* gene.

First, *FUCA1* gene (GenBank id: 2517) primers were designed using MFE primer-3.1 (http://mfeprimer.com/docs/mfeprimer-2.0/) (Additional file 1: Table S1). The PCR amplification of eight exons and intron–exon boundaries of the *FUCA1* gene was carried out in 50 µL containing 50 ng genomic DNA, 0.2 mmol/L dNTPs, 0.4 pmol of each primer, 1.5 mmol/L MgCl_2_, 5% DMSO and 0.5 µL (5 U/µL) Go TaqFlexy (Promega).

Amplification conditions included an initial 5 min denaturation step at 95 °C, followed by 35 cycles of denaturation at 95 °C for 35 s, annealing at 54 °C and extension for 1 min at 72 °C, and finally an extension step for 7 min at 72 °C. The PCR products were purified and then utilized as templates for direct sequencing with the same PCR primers in both forward and reverse directions.

Sequencing was performed at the Laboratory of Biochemistry and Molecular Biology at the Bechir Hamza Children's Hospital, Tunis. The PCR products were purified from excess primers, and dNTP using the FavorPrep KitTM (Favorgen Biotech Corp) and sequenced in both forward and reverse directions using the same PCR primers using the Big Dye Terminator v1.1 Cycle Sequencing Kit (Applied Biosystems, Foster City, CA,USA).The PCR products were purified using Illustra MicroSpin G-50 Columns (GE Healthcare) and electrophoresed on an automated ABI PRISM 310 (Applied Biosystems, Foster City, CA,USA) genetic analyzer and interpreted with the ChromasPro 2.4.1 software (http://technelysium.com.au/wp/chromaspro/).

### Computational analyses

Three online prediction programs (PolyPhen, http://genetics.bwh.harvard.edu/pph2 [10]; pMut, http://mmb.pcb.ub.es/PMut/analyses [11]; and SIFT https://sift.bii.a-star.edu.sg/ [12], were used to predict the pathogenicity of the novel p.G332E missense mutation reported in this study. These programs are based on sequence conservation, differences in amino acid properties, localization of mutated residues in functional sites, and protein stability changes upon missense mutations. On the other hand, the prediction of the impacts of this variant on protein stability was performed using DynaMut tool after the determination of the change in Gibbs free energy (∆∆G) [13].

Additionally, the Human Splicing Finder (HSF) algorithm [14]was used to predict splicing abnormalities generated by the novel splice site mutation c.662+5g>c. This online program (www.umd.be/HSF 3.1) calculates the consensus values (CVs) for mutated and wild-type sequences. For ΔCV values higher than or equal to 10%, the mutation is denoted as significant, on the basis of empirical studies of known splicing mutations.

### Molecular modeling of the FUCA1 protein

The protein sequence for human tissue alpha-l-fucosidase (UniProtKB id: P04066) was retrieved from UniProt to generate a 3D structural model of the FUCA1 protein. The computer-generated model was then constructed by the protein homology modeling server SWISS-MODEL using the crystal structure of the alpha-l-fucosidase enzyme of *Thermotogamaritima* (TM aFuc) (PDB ID; 2zwy) as a template [15]. Building a homology model using SWISS-MODEL server comprises four main steps: (1) structural templates identification, (2) alignment between target sequence and template structures, (3) model-building, and (4) model quality evaluation.

The evaluation of the generated model was determined after the calculation of the GMQE (Global Model Quality Estimation) and QMEAN (Qualitative Model Energy Analysis) scores.

The resulting GMQE score, comprise between 0 and 1, reflects the expected accuracy of the generated model with that alignment and template. It combines properties from the target–template alignment and the template structure. Higher GMQE score indicates higher reliability. The QMEAN Z-score provides an estimate of the “degree of nativeness” of the obtained structure and indicates whether the QMEAN score of the model is comparable to what one would expect from experimental structures. QMEAN Z-scores below − 4.0 are an indication of models with low quality, while those around zero indicate good agreement between the model structure and experimental structures [16].

The novel and previously identified mutations were then localized into a 3D model using Deep View Swiss-Pdb Viewer 4.1 and POV-Ray 3.6 software [17]. Further crystallographic structure analyses were performed to predict the protein stability change upon the reported missense mutation p.G332E.

## Results

### Clinical features and FUCA1 activity

The clinical characteristics of each patient, their leukocyte FUCA1 activities, and identified genotypes are summarized in Table 1.

### FUCA1 mutation analysis

**Fig. 2:**
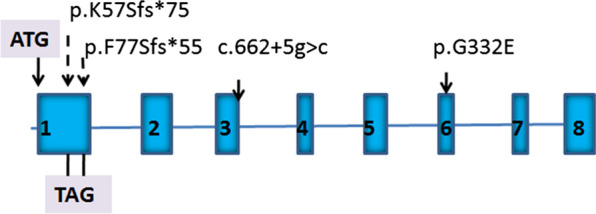
Position of the FUCA1 mutations identified in this study: p.K57Sfs*75, p.F77Sfs*55, p.G332E, and c.662+5g>c

Complete sequencing of the coding and intron–exon junctions was performed for the three unrelated patients suspected of fucosidosis. We identified four novel mutations: two frameshift mutations p.F77Sfs*55 (c.230delT) and p.K57Sfs*75 (c.170delA), one missense mutation p.G332E (c.994G>A), and one splice site mutation (c.662+5g>c) (Fig. 2). None were found in over 50 unrelated individuals.

Additionally, a large number of polymorphisms could be simultaneously identified: c.-13G>A, c.-25T>A, p.P10R IVS1+39C>T, p.L172L; c.516C>T, p.L194N, p.P213P; c.639A>T, p.Y216F; c.647A>T, IVS3-25 T>A, g.2806G>A, c.509+38 c>g, rs180788085, rs907245739, rs1329117558, rs965877153T, rs1344267327, and rs370615681. Ten of them were novel, and seven were previously described (Table 1).

Patient1 (P1) was homozygous for a novel frameshift p.K57Sfs*75 mutation. This novel frameshift mutation in exon 1 produces premature termination of the FUCA1 glycopeptide. The patient (P1) did not have detectable FUCA1 activity and presented with a severe form of the disease (form I). The c.170delA mutation results in the deletion of adenine from the codon (AAG) and produces an aberrant protein with 75 abnormal residues starting from Ser57, giving rise to an aberrant protein lacking 334 amino acids.

Patient 2 (P2), who also presented form I of fucosidosis, was homoallelic for the novel mutation p.F77Sfs*55. This novel frameshift mutation in exon 1 was a thymine deletion at position 230 of the cDNA that led to a change of phenylalanine (TTC) to serine (TCA) at position 77 in the FUCA1 protein, associated with the occurrence of premature glycopeptide truncation of about 334 C-terminal residues.

Patient 3 (P3), who presented form II fucosidosis, was a compound heterozygote for the two novel mutations (c.662+5g>c and p.G332E). c.662+5g>c is a splice site mutation at the donor site of intron 3.The missense mutation (c.994G>A; p.G332E) in exon 6 is a substitution of glycine by glutamate at position 332 of the FUCA1 protein.

### Bioinformatics analysis

Bioinformatics analyses, performed by PolyPhen, predicted that the missense p.G332E mutation was probably damaging, with a score of 0.977. Analysis of pMut and SIFT yielded a probability of a deleterious mutation of 0.82 and 0.00, respectively. The results are summarized in Table 2.

**Table 2:** Predicted effects of the FUCA1 variants

Variants	Exon/Intron	Prediction		
Deletions				
c.170delT; p.K57Sfs*75^a^	Ex 1	Frameshift		
c.230delT; p.F77Sfs*55^a^	Ex 1			
Missense variants		PolyPhen	PMut	SIFT
c.-13G>A^a^	5’UTR			
c.-25 T>A^a^	5’UTR			
rs2070956; c.29C>G; p.P10R	Ex 1	0.272	0.04 (98%°) Neutral	0.17
IVS1+39C>T^a^	Int 1			
rs129756703	Int 1			
p.L172L; c.516C>T^a^	Ex 2			
p.L194N^a^;	Ex 3		0.43 (85%), Neutral	
p.P213P; c.639A>T^a^	Ex 3			
p.Y216F; c.647A>T^a^	Ex 3	0.022		0.08
IVS3+5G>C^a^; C.662+5g>c	Int 3			
IVS3-25T>A^a^	Int 3			
rs180788085C>T	Int 4			
rs907245739	Int 4			
Rs1177361428; p.G332E; c.994G>A^a^	Ex 6	0.977	0.82 (90%), Disease	0.00
Rs1329117558C>T	Int 8			
Rs965877153T>C				
Rs1344267327				
g.2806G>A^a^	5′UTR			

*UTR* untranslatedregion, *Ex* exon, *Int* Intron

^a^Novel

We have highlighted that the c.662+5g>c splice site mutation activates a cryptic acceptor site (TAGgtatga) in intron 3, 86 nucleotides upstream (c. 662+86) with an HSF score of 86.88. This result suggests that the novel splice site mutation in intron 3 of the *FUCA1* gene may cause either intron retention or cryptic splice site utilization.

### Mutational analysis and homology-based modeling of FUCA1 enzyme

The computer generated model of human tissue alpha-l-fucosidase showed good stereo chemical property in terms of overall G-factor value − 0.65. The plot reveals that 98.5% of all residues of the model were found in the most favored regions according to Ramachandran plot analysis. The results of QMEAN analysis generated by SWISS-MODEL were also used to evaluate and validate the model. The GMQE and QMEAN Z-score of the model was 0.622 and − 2.42, respectively, showing the good quality of the model.

**Fig. 3:**
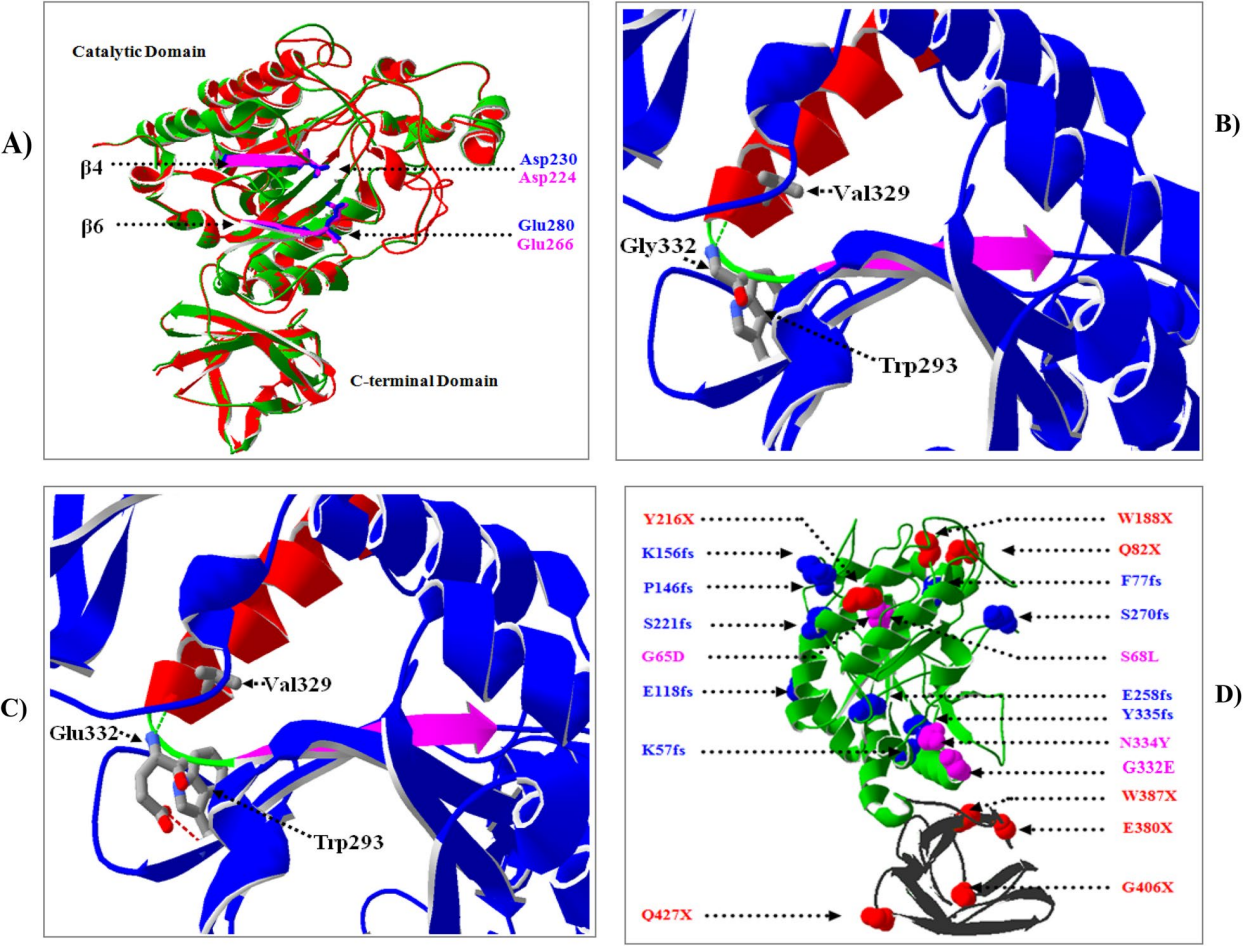
Crystallographic structure analysis of the Human Tissue alpha-l-fucosidase. **A** Structural overlay of the homology model of the Human Tissue alpha-l-fucosidase (green ribbon) with that of the *T. maritima* bacterium (red ribbon—PDB ID; 2zwy). The secondary structure elements carrying the catalytic residues and bound ligands are indicated in blue and pink for Human and T. maritima alpha-l-fucosidase, respectively. **B**, **C** The reported mutation Gly332Glu is located in the loop (colored in green) connecting the seventh strand (colored in pink) with the eleventh helix (colored in red). Gly332 forms one hydrogen bond (shown as green dotted line) with Val 329 (**B**), whereas (**C**) the mutant residue Glu332 is predicted to create a novel hydrogen bond and a steric clash (shown as red dotted line) with the Asp54 residue. The images were prepared using Swiss-PdbViewer 4.1.0 and POV-Ray 3.6 software. **D** The previously reported variants of Human Tissue alpha-l-fucosidase are indicated by dotted arrows. The nonsense, missense, and frameshift mutations are colored in red, blue, and pink, respectively. The catalytic domain is shown in green ribbon, whereas the C-terminal domain is indicated in black. The 3D structure is oriented to best display all previously described mutations

A sequential alignment between the modeled human alpha-l-fucosidase and the alpha-l-fucosidase from the marine hyperthermophilic bacterium *Thermotoga maritima* (Tm α fuc) showed that both protein sequences shared only 38% identity. In contrast, the structural superposition of both molecules showed a highly conserved 3D structure (Fig. 3A).The derived model of the catalytic domain of human alpha-l-fucosidase is highly similar to that of TM aFuc and thus reflects the high level of sequence and structural conservation found between these two enzymes [18].

The constructed tertiary structure of the human tissue alpha-l-fucosidase showed that the FUCA1 protein was composed of two domains: a catalytic domain in the N-terminal amino acids and a C-terminal domain (Fig. 3A). The catalytic domain adopts an (α/β)8 barrel-like fold, with eight parallel strands localized in the central axis and surrounded by six α-helices. The secondary structure elements carrying the catalytic residues (Asp225 and Glu275) and bound ligands are located at the end of the fourth and the sixth β-strands. The secondary structure of the C-terminal domain is constructed of eight antiparallel strands packed into two β-sheets forming a two-layer β-sandwich.

In this study, the novel missense mutation p.G332E was found to be located near a catalytic nucleophilic residue buried in the loop connecting the seventh β-strand with the eleventh α-helix (Fig. 3B, C). The Gly332 residue is implicated in the formation of a hydrogen bond with Val329.In contrast, the mutant (Glu332) contains a negatively charged amino group on its side chain, which is often involved in forming H-bonds with positively charged residues compared to the wild-type. The introduced charge at this position can cause repulsion between the mutant residue and neighboring residues. In the presence of the missense mutation p.G332E, the loop will be disturbed after the creation of a steric clash between the mutant and Trp293 backbone buried in the sixth β-strand. Furthermore, the torsion angles for this large residue (Glu332) are unusual. Only glycine is flexible enough to make these torsion angles, and mutation into another residue will force the local backbone into an incorrect conformation and disturb the local structure. The reported mutation introduces an amino acid with different properties that can destabilize the conserved structure (ΔΔG: − 2.517 kcal/mol as predicted by DynaMut tool) shared by both humans and *Thermotoga maritima* alpha-l-fucosidase (Tm α fuc).

## Discussion

Fucosidosis is a rare autosomal recessive lysosomal storage disease caused by α-l-fucosidase deficiency due to *FUCA1* gene mutations. Its estimated frequency is below 1 in 200,000 live births, depending on the country [6].

This study was the continuation of the largest Tunisian survey of fucosidosis patients diagnosed during 1987–2007. In Tunisia, the real frequency of this disease is underestimated, considering the significant number of suspected cases through pedigree analysis of affected families [19].

According to the age of onset and the degree of severity, the phenotypic expression of fucosidosis includes two phenotypes: severe (type I or form I) and milder (type II or form II) [1]. The diagnosis of fucosidosis patients was based on the characteristic pattern of urinary oligosaccharides and an enzymatic assay in leukocytes in the studied patients. Based on clinical examination, two patients (P1 and P2) were classified as type I with a severe disease, while one patient (P3) was classified as type II with a milder disease.

According to the clinical data, the confirmation of the diagnosis in all patients with fucosidosis was performed at the mean age of two years, as described in the literature [20].

All patients presented with an early onset of psychomotor retardation within the first year of life and developed severe spastic quadriplegia. Severe growth retardation was noted in all patients, and all cases presented variable degrees of dysostosis multiplex on radiological investigations.

In this study, angiokeratoma was observed in the patients (P3 and P1) with type I and type II fucosidosis, respectively. The clinical profile of the patients (P3 and P1) was in agreement with several studies described in the literature [20]. Of note, angiokeratomas do not represent a pathognomonic criterion since this phenotypic description is present in other pathologies, such as Fabry disease and sialidosis [21].The presence of angiokeratoma has been detected in patients with type I who develop faster neurological deterioration leading to early death [20].

Cases of both patients (P1 and P2) were classified as type I disease. Nevertheless, only patient P1 developed a faster neurological deterioration that led to an earlier death compared to patient P2 and P3.

To the best of our knowledge, we have described the first molecular analysis of *FUCA1* in three unrelated patients with fucosidosis. The genotypes of the patients were: p.F77Sfs*55/p.F77Sfs*55, p.K57Sfs*75/p.K57Sfs*75, and p.G332E /c.662+5g>c.

With regard to the pathogenicity of the novel mutations, the frameshift mutations caused by a single base deletion (p.F77Sfs*55 and p.K57Sfs*75) are located in the glycoside hydrolase catalytic domain of the FUCA1 protein and are both predicted to introduce premature termination of glycopeptides in which the amino acids of the downstream sequence are completely altered. Although the functional test was not further characterized in this study, the two frameshifts, p.K57Sfs*75 and p.F77Sfs*55, were identified in patients P1 and P2, respectively, who did not have detectable FUCA1 activity, which was consistent with the severe observed phenotype. Furthermore, clinical variability was observed in the two patients (P1 and P2) with the two frameshift mutations. The phenotypic heterogeneity seemed to be secondary to unknown factors [7, 22].

The third novel alteration, p.G332E, was a missense mutation, probably involving damage to protein function, based on the PolyPhen-2prediction algorithm. Additionally, we found that the missense mutation occurred in the conserved domain among human lysosomal sulfatases, and the conserved domains among sulfatases have been known to be essential for the catalytic activity [23]. The p.G332E mutation associated with the novel splice site mutation (c.662+5g>c) has been identified in patient P1.The combination of the c.662+5g>c variant of the missense mutation p.G332E allows the patient (P1) to present a milder phenotype.

The structure of human FUCA1 was modeled by homology using the X-ray crystal structure of the bacterium Tm α fuc [15, 24, 25]. The structural prediction analysis that the Gly332 residue is involved in the formation of an extremely structured loop that serves to reverse the direction of the seventh β-strand polypeptide to the eleventh α-helix. Additionally, the p.G332E mutation is located close to the secondary structure of elements carrying the catalytic residues buried at the end of the fourth and the sixth β-strands. Thus, this mutation could prevent the normal folding of the protein as well as its function. In the literature, only one missense p.N329Y mutation has been identified in this conserved loop in the homozygous form [8]. The p.N329Y genetic lesion has already been identified in an Australian patient presenting with a severe phenotype. Gly332 is located near the active site of FUCA1 in a conservative region, suggesting the severity of the mutation. The combination of c.662+5g>c and the missense mutation p.G332E provided patient P1 with a milder phenotype. Consequently, the fourth novel mutation, c.662+5g>c, may provide enough residual activity to avoid a severe phenotype. Of note, only one donor splice site mutation c.954+1G>A identified in intron 5 was detected in a homozygous status in an East Indian-Zambian patient who developed a severe form of fucosidosis [26]. Furthermore, several studies have shown that donor splice site mutations are generally more prevalent than the acceptor splice site variants [26].

Interestingly, the 3D structure analyses have demonstrated that the novel identified mutations (p.F57fs, p.K77fs, and p.G332E) and most of the reported mutations (p.G65D, p.S68L, p.Q82X, p.146fs, p.K156fs, p.E118fs, p.W188X, p.N334, p.E258fs, p.S270fs, p.Y335fs, and p.Y216X) are located in the catalytic domain of the FUCA1 protein (Fig. 3D). These are mainly frameshift variations which affect the helices surrounding the central axis of this catalytic domain. Moreover, among the observed missense mutations, four were close to the catalytic sites, and three nonsense mutations were located on the sides. However, only four nonsense mutations (p.E380X, p.387X, p.G402X, and p.G427X) have been identified in the C-terminal domain of the FUCA1 protein [27] (Fig. 3D).

In addition to these mutations, a large number of FUCA1 sequence variations were identified in the Tunisian fucosidosis alleles (Table 2). The noncoding variations (rs180788085, rs907245739, rs1329117558, rs965877153T, and rs1344267327) and coding (p.P10R, p.L172L, p.L194N, p.P213P, and p.Y216F) polymorphisms/sequence variants do not have effect on the clinical phenotypic severity of fucosidosis. Indeed, such polymorphisms were detected in patients with severe (P1 and P2) and milder (P3) phenotypes.

However, several findings support the notion that polymorphisms of many genes may play a role in the pathophysiology of a major disease, such as infectious diseases in tuberculosis, and leishmaniasis [28, 29], and in diabetes [30]. A large variability in clinical responses is observed, which justifies the crucial role of SNPs in the pathophysiology of these diseases, especially when the polymorphisms are located in the promoter regions [31]. We hypothesize their participation in the regulation of molecular mechanisms.

The characterization of these mutations aims to elucidate the allelic heterogeneity of fucosidosis phenotypic aspects, thereby providing more information on the impact of the mutant residues on the FUCA1 structure. These findings will be of importance in the development of new approaches for therapies in patients with fucosidosis.

The continuous characterization of novel molecular defects responsible for defective alpha-l-fucosidase (FUCA1) activity helps to better understand the molecular mechanisms of enzyme secretion, function, and interaction with substrate or other proteins. On the other hand, in silico analysis helps to provide comprehensive insight into molecular mechanisms of biological processes after the determination of the impact of the identified mutations on structure or enzyme functionality. Understanding of the mutational effects on protein stability is essential for optimizing the expression, purification, storage and formulation of proteins in biotechnology and pharmaceutical industries.

## Conclusion

While the results obtained by bioinformatics algorithms can only be predictive and need to be confirmed by subsequent functional studies, they can be used as real tools to define the possible impact of a mutation identified in relation to the pathology observed (in this case, fucosidosis). They allow a better understanding of these molecular disorders, thereby providing a better understanding of the genotype/phenotype correlation. Moreover, these molecular data will allow for accurate carrier detection, prenatal diagnosis, and counseling for fucosidosis disease in Tunisia, where first-cousin consanguineous marriage remains frequent.

## Supplementary Information

**Additional file 1:** Table S1; List of primers used in PCR reactions and sequencing.

## Data Availability

The datasets used and analyzed during the current study are available from the corresponding author upon request. The mutations of the fucosisdosis patients were submitted to ClinVar database (https://www.ncbi.nlm.nih.gov/clinvar/) under Accession Numbers VCV000979023, VCV000979026, VCV000979024 and VCV000979025.

## Abbreviations

FUCA1: Alpha-l-fucosidase
HSF: Human splicing finder
TM aFuc: *Thermotoga maritima* Alpha-l-fucosidase enzyme
