# Correction to: Fucosidosis in Tunisian patients: mutational analysis and homology-based modeling of FUCA1 enzyme

**DOI:** 10.1186/s12920-021-01149-w

**Published:** 2021-12-15

**Authors:** Latifa Chkioua, Yessine Amri, Sahli Chaima, Ferdawes Fenni, Hela Boudabous, Hadhami Ben Turkia, Taieb Messaoud, Neji Tebib, Sandrine Laradi

**Affiliations:** 1 Research Laboratory of Human Genome and Multifactorial Diseases, Faculty of Pharmacy, University of Monastir, Street Avicenne, 5000 Monastir, Tunisia; 2 Biochemistry Laboratory (LR 00SP03), Bechir Hamza Children’s Hospital, Tunis, Tunisia; 3 Pediatrics Department, La Rabta Hospital, Tunis, Tunisia; 4 The Auvergne-Rhône-Alpes Regional Branch of the French National Blood System EFS/GIMAP-EA 3064, 42100 Saint Etienne, France

## Correction to: BMC Med Genomics (2021) 14:208 https://doi.org/10.1186/s12920-021-01061-3

Following publication of the original article [1], the authors identified an error in the author name of Sahli Chaima.

The incorrect author name is:

Chayma (given name) Saheli (family name)

The correct author name is:

Sahli (given name) Chaima (family name)

The author group has been updated above and the original article [1] has been corrected.
