# Supplementary material for: Fucosidosis in Tunisian patients: mutational analysis and homology-based modeling of FUCA1 enzyme
Source: BMC Med Genomics. 2021 Aug 23;14:208. doi: 10.1186/s12920-021-01061-3 (PMC8383439; doi:10.1186/s12920-021-01061-3)
Supplement: Supplementary file 1 — Additional file 1: Table S1; List of primers used in PCR reactions and sequencing. [file 12920_2021_1061_MOESM1_ESM.docx]

| **Number of exon** | Primer name | Sequence 5′ > 3′ |
| --- | --- | --- |
| **Exon1** | F1 FUCA1 | 5’GCCAATCGTTAGTCAGAGTG 03’ |
|  | R1 FUCA1 | 5’CCACTGATTTGAAACCCTGG3’ |
| **Exon2** | F2 FUCA1 | 5’GGCATGCTGGGCAAGTTCAT3’ |
|  | R2 FUCA1 | 5’AGGAGTAGAAGGCCAAAAGC3’ |
| **Exon3** | F3 FUCA1 | 5’AGTAAACCTCATTTTGCTTA 3’ |
|  | R3 FUCA1 | 5’GAAGCTCTGATTTCATTGAT3’ |
| **Exon4** | F4 FUCA1 | 5’GCTGCTGGACTCAAGCTTAG3’ |
|  | R4 FUCA1 | 5’ACTCCAGAGTTTGGCTCCTT3’ |
| **Exon5** | F5 FUCA1 | 5’AAAGCTTGCAAAAACAGCAT3’ |
|  | R5 FUCA1 | 5’GGTAGAGACCAGGGATGTTA3’ |
| **Exon6** | F6 FUCA1 | 5’ATTAAAACAGCAGAGGATAT3’ |
|  | R6 FUCA1 | 5’CAAATAGGGGTACAAATTTTA3’ |
| **Exon7** | F7 FUCA1 | 5’CAATGGCTCATACTTGTAAT3’ |
|  | R7 FUCA1 | 5’GTCTAAAAGAGGTGTGAATA3’ |
| **Exon8** | F8 FUCA1 | 5’CCTCTTTTAGACTGGCT TTC3’ |
|  | R8 FUCA1 | 5’CACACAAACCAGAAACTGAT3’ |

Table S1: List of primers used in PCR reactions and sequencing.
